# Validation of western blot for *Histoplasma capsulatum* antibody detection assay

**DOI:** 10.1186/s12879-016-1427-0

**Published:** 2016-02-24

**Authors:** Marcos de Abreu Almeida, Cláudia Vera Pizzini, Lisandra Serra Damasceno, Mauro de Medeiros Muniz, Rodrigo Almeida-Paes, Regina Helena Saramago Peralta, José Mauro Peralta, Raquel de Vasconcelos Carvalhaes Oliveira, Alexandre Gomes Vizzoni, Carla Lourenço Tavares de Andrade, Rosely Maria Zancopé-Oliveira

**Affiliations:** Instituto Nacional de Infectologia Evandro Chagas, Fundação Oswaldo Cruz, Avenida Brasil, 4365, Manguinhos, Rio de Janeiro, RJ Brazil; Departamento de Patologia, Universidade Federal Fluminense, Rua Marquês de Paraná, 303, Centro, Niterói, RJ Brazil; Instituto de Microbiologia Prof. Paulo de Goes, Universidade Federal do Rio de Janeiro, Avenida Carlos Chagas Filho, Cidade Universitária, Ilha do Fundão, Rio de Janeiro, RJ 373 Brazil; Escola Nacional de Saúde Pública, Fundação Oswaldo Cruz, Avenida Brasil, Manguinhos, Rio de Janeiro, RJ 4365 Brazil

**Keywords:** Histoplasmosis, Immunodiagnostic, Western blot, Sensitivity, Specificity

## Abstract

**Background:**

Histoplasmosis is worldwide systemic mycoses caused by the dimorphic fungus *Histoplasma capsulatum*. The isolation and identification of *H. capsulatum* in culture is the reference test for histoplasmosis diagnosis confirmation. However, in the absence of it, serology has been used as a presumptive diagnosis through antibody and antigen detection. The purpose of the present study was to validate an immunoassay method (western blot) for antibodies detection in the diagnosis of histoplasmosis.

**Methods:**

To validate the western blot (WB) a study was conducted using 118 serum samples from patients with histoplasmosis and 118 serum controls collected from January 2000 to December 2013 in residents of the Rio de Janeiro State, Brazil. Diagnostic validation parameters were calculated based on the categorization of results obtained in a 2 × 2 table and subjected to statistical analysis. In addition, the viability of deglycosylated histoplasmin antigen (ptHMIN) onto nitrocellulose membranes previously sensitized was evaluated during the same period.

**Results:**

The WB test showed sensitivity of 94.9 %, specificity of 94.1 %, positive predictive value of 94.1 %, negative predictive value of 94.9 %, accuracy of 94.5 %, and almost perfect precision. Besides, the strips have proved to be viable for using at least 5 years after ptHMIN antigen sensitization.

**Conclusion:**

Western blot test using ptHMIN provides sensitive, specific, and faster results. Therefore, could be considered a useful tool in the diagnosis of histoplasmosis being used by public health system, even in situations where laboratory facilities are relatively limited.

## Background

Histoplasmosis is a systemic disease caused by the dimorphic fungus *Histoplasma capsulatum*. This disease has a worldwide distribution and is one of the most common respiratory mycosis, presenting endemic areas in certain regions of the United States and Latin America [1, 2]. In Brazil, these regions are located throughout the country, especially in the Midwest, Northeastern and Southeast regions [3, 4]. The infection is acquired by inhalation of fungal infectious propagules present in organic matter rich soil, mainly with excreta of birds and bats [1].

The clinical spectrum of this illness ranges from asymptomatic, self-limited illness to a progressive disseminated disease. Although the clinical manifestations of histoplasmosis are well described, there is significant overlapping of symptoms with other diseases, and the diagnosis cannot be achieved based on clinical information alone. [5]. Microbiological diagnosis is based on isolation of the fungus in cultures, and microscopic examination of fluids and tissues using specific staining techniques [5]. Nonetheless, these methods have limitations. Culture examination is slow, taking up to 2–4 weeks, and requires level 3 biosafety facilities [6]. The sensitivity and specificity of histopathological examination vary depending on the patient’s clinical status, exhibiting reduced sensitivity in the subacute and chronic forms of the pulmonary histoplasmosis [6, 7]. In addition, the specific role of each test fluctuates according to the clinical form, since variations in sensitivity have been associated to different clinical presentations.

Serological methods usually have a rapid turnaround time and detection of either antibodies or antigens can provide information indicative of current disease. Historically the identification of antibody responses in patients with histoplasmosis has proved useful in the diagnosis of disease since serological evidence is the prime diagnostic indicator of histoplasmosis [8].

Serological diagnosis for histoplasmosis focuses on the identification of antibodies to the H [9] and/or M antigens [10]. The two routine antibody detection methodologies are complement fixation (CF) and immunodiffusion (ID), because of convenience, availability, and accuracy of these assays. In the past, CF was a popular test to diagnose histoplasmosis but ID has been found to be more specific [3]. The ID test qualitatively measures precipitating antibodies, and is specific for the detection of antibodies to M and H antigens, ranging from 70 to 100 % [11]. However, it has low sensitivity, mainly in acute, disseminated and opportunistic manifestations of the disease. In general, ID is useful for detecting antibodies 4–6 weeks after infection with *H. capsulatum*. Detection of precipitins by immunodiffusion is one of the most widely available techniques for diagnosis [3], and although the presence of immunodiffusion bands are less reliable than culture, anti-Histoplasma antibodies may be detected in the serum of 90 % of patients with histoplasmosis [12]. The presence of M and H bands is highly suggestive of active Histoplasma infection [8]. Several enzyme-linked immunosorbent assays (ELISA) protocols have been described for antibody detection in histoplasmosis using diverse antigenic preparations showing sensitivity among 75 to 100 % [13–16].

The reference protocols for the evaluation of the diagnosis of infectious diseases show that the diagnostic target must first be identified, followed by the optimization of reagents and test used. Then, the performance of the method should be evaluated [17]. In the past, a WB test was developed for detection of antibodies against native glycosylated and chemically deglycosylated M and H antigens of *H. capsulatum* in serum obtained from patients during the acute phase of pulmonary histoplasmosis showing 90 and 100 % sensitivity, for the acute and convalescent-phase respectively, and 100 % specificity [18]. This test met the requirements of a good diagnostic test for the acute and convalescent-phase of histoplasmosis. The advantage of the WB test in relation to routine serology is the identification of some cases early in infection, before seroconversion can be detected by CF and ID, showing a high degree of sensitivity and specificity, Also, this methodology is faster and easier to realize than those tests used in diagnostic routine. However, these immunoassays were used only in acute and proven histoplasmosis cases [16, 18], and it would be very important validate this methodology for diagnosis of several forms of histoplasmosis in a large number of cases, since it could be used in conjunction with culture to improve the diagnosis of *H. capsulatum* infection particularly in cases when microorganism isolation procedure is negative, and also guide for the specific therapy [5].

Our aim in this study was to validate this western blot immunoassay to detect antibodies in the serodiagnosis of histoplamosis following the reference protocols for the evaluation of diagnostic tests for infectious diseases in order to determine the diagnostic accuracy of this test. In addition, the antigenic reactivity of deglycosylated histoplasmin antigen (ptHMIN) onto nitrocellulose membranes previously sensitized was evaluated during the same period as a secondary objective of this study.

## Methods

### Target population and histoplasmosis case definition

**Fig. 1:**
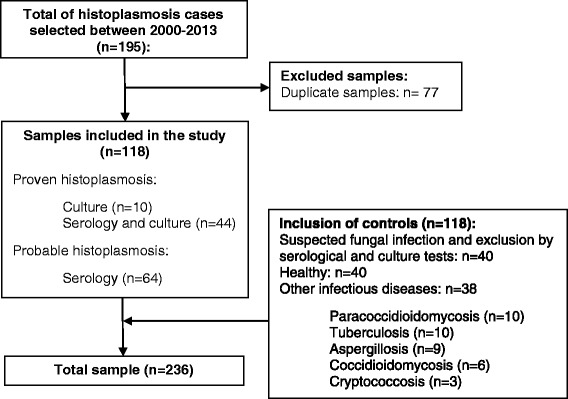
Serum samples tested during validation Western blot immunoassay for diagnosis of histoplasmosis

A study was conducted in a total of 236 serum samples collected from January 2000 to December 2013 at the Instituto Nacional de Infectologia Evandro Chagas (INI), Fiocruz from residents of Rio de Janeiro State without prior treatment for histoplasmosis. The target population was separated into two groups, histoplasmosis group and a control group. The histoplasmosis group (*n* = 118) was comprised of patients with epidemiological and clinical history compatible with histoplasmosis. The diagnosis criteria were based on a positive result in culture, or *Histoplasma* antibody detection demonstrating H or M precipitin bands by immunodiffusion [19]. The control group was obtained based on the proportion 1:1 case–control, a total of 118 individuals of the same population. The serum samples of control group were randomly obtained from 40 patients with clinical suspicion of fungal infection, but excluded of the diagnosis criteria selected, 28 patients suspected of other pulmonary mycosis (paracoccidioidomycosis, *n* = 10; aspergillosis, *n* = 9; coccidioidomycosis, *n* = 6; and cryptococcosis, *n* = 3); 10 patients with tuberculosis; and 40 healthy blood donors (Fig. 1).

The definition of histoplasmosis was based on the consensus EORT/MSG [20], and the clinical forms followed recommendations [7, 21]. Proven histoplasmosis included individuals with identification of *H. capsulatum* yeasts in cultures or histopathological analyzes. Positive ID combined with clinical and radiologic findings were required for classification of probable disease. All individuals included in the study were probed to detect antibodies against *H. capsulatum* by WB and ID tests.

### Study design

This is a retrospective study based on standard clinical, laboratorial and epidemiological dates collected in the medical records of INI/Fiocruz.

The variable analyzed were age, gender, comorbidity (AIDS, tuberculosis), specific laboratory tests as serology by ID, mycological tests and clinical form of illness.

Clinical and laboratory data were collected by an independent investigator, blinded to clinical information.

### Ethical statement

This study was approved by the Research Ethics Committee of the Instituto Nacional de Infectologia Evandro Chagas, Fiocruz, accession number 19109913.0.0000.5262.

### Serologic tests and antigens

ID tests to detect antibodies to histoplasmin (HMIN), paracoccidioidin and *Aspergillus fumigatus* antigen were performed on serum specimens obtained from all individuals enrolled in this study [22]. HMIN was produced from mycelium-form cultures of *H. capsulatum* IGS 4/5 (INCQS 70308) as described previously [23], and H and M antigens were chromatographically purified [24]. Chemical deglycosylation was achieved according to previously studies [25, 26]. Briefly, sodium *meta*-periodate (NaIO_4_) was added to 2-ml aliquots of purified antigens to a final concentration of 100 mM. After incubation for 18 h at 4 °C in the dark, residual periodate was consumed by incubation for 15 min with an equimolar amount of glycerol, followed by addition of 1 M sodium borohydride. After incubation for 2 h at 4 °C, the reaction mixture was dialyzed against cold deionized water.

### Western blot (WB) immunoassay

Native and deglycosylated antigens were analyzed by sodium dodecyl sulfate-polyacrylamide gel electrophoresis (SDS-PAGE), on 10 % polyacrylamide resolving gels with a 4 % polyacrylamide staking gel. The gels were then processed for western blot performed according to the protocol previously established [18]. Briefly, after electro transfer of proteins (16.75 ug) to nitrocellulose membranes in a Mini Trans Blot cell (Bio-Rad), membranes were sliced vertically, and free binding sites in the membranes were blocked by incubation for 60 min in 5 % (wt/vol) nonfat milk in 20 mM Tris–HCl–500 mM NaCl–0.2 % Tween 20 (pH 7.5) (TTBS). Next, strips were incubated for 60 min at room temperature with serum specimens diluted 1:100 in TTBS containing 5 % nonfat milk. Strips were washed in TTBS three times for five minutes each; and then goat anti-human immunoglobulin G (IgG)-alkaline phosphatase conjugates (Jackson ImmunoResearch, EUA) diluted in TTBS (1:3.000) were added and incubated as described above. Following incubation, blot strips were washed and incubated with substrate solution consisting of 5-bromo-4-chloro-3-indolylphosphate (BCIP; 15 mg/ml in dimethylformamide [DMF]) and nitroblue tetrazolium (NBT; 30 mg/ml in 70 % aqueous DMF). Substrate stock solutions were diluted 1:100 before use in Tris/NaCl buffer (100 mM Tris–HCl [pH 9.5], 100 mM NaCl, 50 mM MgCl_2_). After color development strips were rinsed exhaustively in deionized water.

Two experienced investigators with longstanding experience in the WB performed the analysis in the anonymous sera, blindly with regard to the clinical and laboratorial findings. The samples were identified with sequential numbering by a third investigator avoiding the identification of cases and controls by the executors of the analysis. The immunoassays were then carried out separately for each of the two investigators, including all steps of the methodology. The test was repeated three times with readings performed by two investigators in order to evaluate the intra and interobserver agreement index.

### Western blot performance characteristics

To calculate the performance characteristics of the test, the true-positive (TP), false-positive (FP), false-negative (FN), and true-negative (TN) results were analyzed [27].

### Analysis of the membranes

Nitrocellulose membranes previously sensitized with the antigen ptHMIN, lot # Hc05 were maintained at room temperature and tested progressively for the antigenic reactivity over the time periods of 1 day, 1 month, 6 months, 1, 2, 3, 4 and 5 years, by WB with serum sample (#20965) obtained from a proven histoplasmosis case to assess the viability of the membranes containing ptHMIN, respecting the conditions listed above.

### Statistical analysis

The epidemiological and laboratorial data were evaluated by bivariate analysis using Chi-squared test or Fisher exact test if any value in the cells of the contingency table was less than five. Results were considered statistically significant when *p* < 0.05. The diagnostic accuracy of the WB was evaluated by sensitivity, specificity, predictive values and likelihood ratios, with ranges of 95 % confidence intervals (CI). The reproducibility of the test was calculated and classified using agreement index by Kappa coefficient according to Landis & Koch (1977) [28]. In addition, we used odds ratio (OR) to measure the presence of H and M bands simultaneously in the laboratorial tests applied in this study. All data were subjected to statistical analysis by using of Statistical Package for Social Sciences SPSS software, version 16.0.

## Results

### Baseline characteristics of patients

Between January 2000 and December 2013, 118 cases of histoplasmosis were diagnosed in the Instituto Nacional de Infectologia Evandro Chagas, and could be filled on the criteria of inclusion for this study (Fig. 1). The mean age of all patients (cases and controls) was 41.5 years (10-81 years), 75.4 % of the patients were male and 24.6 % female. Among this population, 37.3 % were HIV-patients (Table 1).

**Table 1:** Baseline characteristics histoplasmosis cases (*n* = 118) and control group (*n* = 118)

Variables	Cases n° (%)	Controls n° (%)	*p* value**
Sex			
Men	89 (75.4)	90 (76.3)	1.000
Woman	29 (24.6)	28 (23.7)	
Age (years)			
<40	57 (48.3)	51 (43.2)	0.513
≥40	61 (12.7)	67 (11.0)	
HIV status			
Positive	44 (37.3)	22 (18.6)	0.002
Negative	21 (17.8)	59 (50.0)	
Non-tested	53 (44.9)	37 (31.4)	
Tuberculosis^a^			
Present	20 (16.9)	17 (14.4)	0.720
Absent	43 (36.4)	30 (25.4)	
Missing	55 (46.6)	71 (60.2)	

^a^Diagnosis by isolation of *Mycobacterium tuberculosis* in sample of various clinical specimens; **p value < 0.05

The clinical form most commonly found in all patients with histoplasmosis was pulmonary (50.8 %), followed by disseminated (36.5 %) and mediastinal histoplasmosis (4.2 %). Fifty-four patients had proven histoplasmosis. In the proven cases of histoplasmosis the pathogen was obtained by culture isolation in 60 clinical samples such as bone marrow (*n* = 15), respiratory secretion (*n* = 14), skin lesions (*n* = 9), blood (*n* = 8), mucosal lesions (*n* = 8) and lymph node aspirate (*n* = 6). It must be emphasized that six patients had the fungus isolated from two different clinical samples. In addition, sixty-four were classified as probable histoplasmosis (40 acute pulmonary, 6 chronic pulmonary, 5 disseminated, 3 mediastinal, and 10 non-defined clinical and serological suspected cases who could not be classified in none of the preceding forms) [7, 21] based on the presence of M and/or H bands in immunodiffusion in conjunction with clinical and radiologic findings.

### Immunodiffusion test

All the samples from patients with histoplasmosis were re-tested by ID for the detection of anti-*H. capsulatum* antibodies. Of 118 sera from patients with histoplasmosis, 103 (87.3 %) had reactivity, characterized by the presence of at least one line of precipitation. Fifteen samples (12.7 %) were negative. The results were compared with those performed immediately after blood collection, demonstrating excellent agreement (Kappa = 0.96). None of the 118 control sera that were probed for histoplasmosis, paracoccidioidomycosis, and aspergillosis, were prior positive by this method.

### Western blot

**Fig. 2:**
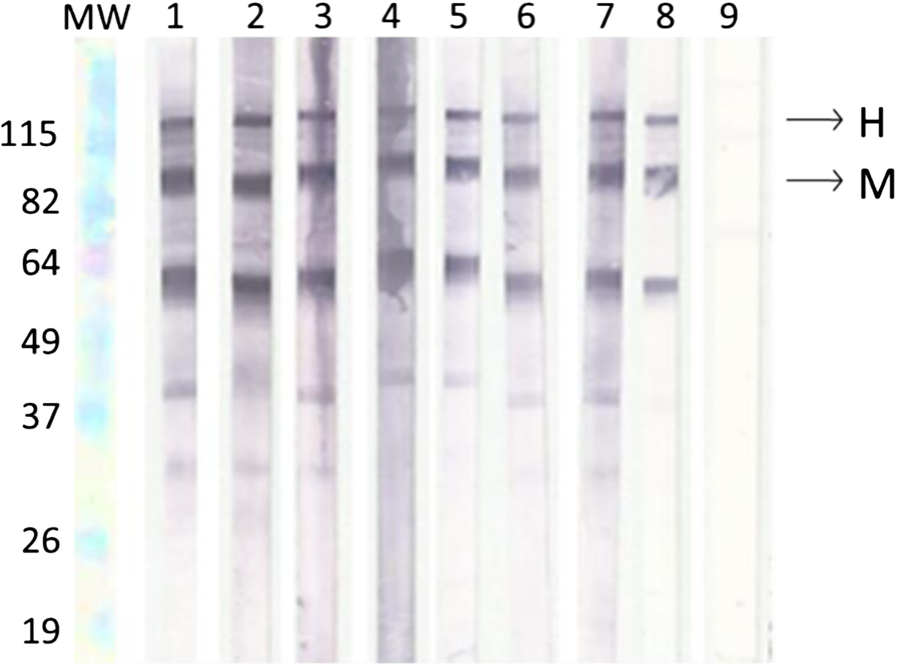
Analysis of the viability of membranes. Lanes MW- Molecular weight; 1- One day; 2- One month; 3- Six months; 4- One year; 5- Two years; 6- Three years; 7- Four years; 8- Five years; 9- Healthy individual (negative control)

Any well-defined band, with molecular weight of 115 and 88 kDa represent the specific antibodies against H and M antigen, respectively (Fig. 2). H and M bands were identified in sera of 47 patients with histoplasmosis (39.8 %). In 65 samples (55.1 %) it was verified just the M band and in six patients (5.1 %) no band was identified.

### Diagnostic Accuracy of the WB for anti-*H. capsulatum* antibodies detection

The analysis of 118 serum samples from proven and “probable histoplasmosis” showed that 112 were correctly classified as true-positive samples and only six samples had false-negative results. These samples were from HIV-patients with disseminated histoplasmosis. Of the 118 control samples, false-positive results were observed in seven samples. Cross-reactivity has seen in sera from patients with paracoccidioidomycosis (5), aspergillosis (1) and tuberculosis (1).

The results of the diagnostic accuracy and their confidence intervals are shown in Table 2. The WB had a sensitivity of 94.9 % (95 % CI: 92.1 to 97.7 %), a specificity of 94.1 % (95 % CI: 91.1 to 97.1 %), an accuracy of 94.5 % (95 % CI: 91.6 to 97.4 %), a positive predictive value (PPV) of 94.1 % (95 % CI: 91.1 to 97.1 %), and a negative predictive value (NPV) of 94.9 % (95 % CI: 92.1 to 97.7 %).

**Table 2:** Serological parameters for the Western blot using ptHMIN

Parameter	Values (%)	CI^a^ 95 %
Sensitivity	94.9	92.1–97.7
Specificity	94.1	91.1–97.1
Accuracy	94.5	91.6–97.4
PPV^b^	94.1	91.1–97.1
NPV^c^	94.9	92.1–97.7
Positive likelihood ratios	16.0	-
Negative likelihood ratios	0.1	-

^a^(*CI*) confidence interval; ^b^(*PPV*) positive predictive value; ^c^(*NPV*), negative predictive value

The reproducibility of the test has shown that the intraobserver and interobserver agreement (Kappa = 0.99 and 0.96, respectively) were classified as almost perfect.

Differently from ID; where both H and M precipitin bands were observed just in 8.9 % of the positive samples tested, in WB the H and M were detected simultaneously in 42.0 % of samples, showing a gain in sensitivity (Table 3).

**Table 3:** Performance results of serological tests for detection of antibodies against *Histoplasma capsulatum*

Histoplasmosis	Immunodifusion (ID)^a^		Western blot (WB)^a^		*p*-value**	OR
	H and M bands	M band	H and M bands	M band		
Proven (*n* = 54)	8	36	21	27	0.009	3.88
Provable (*n* = 64)	2	62	26	38	0.000	21.21
Total (*n* = 118)	10	98	47	65	0.000	7.08

^a^Cases non reagent: ID – 10 cases; WB – 6 cases; **p-value < 0.05 (Chi-square Test and Fisher Exact Test)

### Analysis of the membranes

The evaluation of the viability of previously sensitized nitrocellulose membranes with the antigen ptHMIN was performed by WB probed against the serum sample # 20965 from proven histoplasmosis patient. It was demonstrated reactivity on the membranes up to 5 years (Fig. 2).

## Discussion

The definitive diagnosis of histoplasmosis is usually based on the isolation and identification of the *H. capsulatum* in cultures or by identification of fungi in biological samples with special staining [7, 29]. However, this process is time-consuming and has limitations in sensitivity [30], mainly in acute and disseminated histoplasmosis that need be rapidly diagnosed for the prompt initiation of therapy [31].

Presently, there are additional diagnostic tools available for diagnosis of histoplasmosis to supplement culture and microscopic examination. These laboratory tests have a rapid turnaround time and reasonable specificity and sensitivity. For instance, serological techniques involving antibody and antigen detection have been developed using different methodologies. Immunoassays for antibody detection using ptHMIN, has played an important role in the diagnosis of histoplasmosis showing high sensitivity and specificity for diagnosis of histoplasmosis. Nevertheless, these immunoassays were used only in acute forms and proven histoplasmosis cases [16, 18].

In the past, a WB test was developed for detection of antibodies against native glycosylated and chemically deglycosylated M and H antigens of *H. capsulatum* in serum obtained from patients during the acute phase of pulmonary histoplasmosis showing 90 and 100 % sensitivity, for the acute and convalescent-phase respectively, and 100 % specificity [18]. These results can be compared with the values of sensitivity (94.4 %) and specificity (94.1 %) obtained in this study. However, in the latter it was evaluated serum samples from other clinical forms of histoplasmosis as well from individuals with other fungal infections, with tuberculosis besides healthy individuals, which could explain the small differences found in the sensitivity and specificity parameters. In addition, the results presented here demonstrate the utility of this methodology in the diagnosis of histoplasmosis through more robust evaluation in different populations being faster than those test used in diagnostic routine, and should be applied in microbiology laboratories since has almost perfect reproducibility, producing repeated and consistent results.

The WB evaluated here showed to be an excellent diagnostic test for histoplasmosis since it fits all criteria suggested by international guides for accuracy in testing such as high sensitivity and specificity; ease to use and storage [17, 32]. Then, this is the first description about WB validation using ptHMIN for diagnosis of several forms of histoplasmosis in a large number of cases. Serological histoplasmosis diagnosis is focused on the identification of anti-H and anti-M antibodies by ID and the presence of both the H and M precipitins in a serum sample is considered to be conclusive for the diagnosis of this mycoses [3]. However, the M band is mostly found in acute and chronic forms of histoplasmosis. This band persists for months to years after the infection has resolved [30], and the H band is present only in 7.0 % of sera from patients with acute histoplasmosis, and rarely can be found without M band. The presence of H band is indicative of chronic or severe acute forms of histoplasmosis [33]. In this study, the presence of both anti-M and anti-H antibodies was more frequently observed in WB than ID, in both proven and probable cases of histoplasmosis due the higher sensitivity of primary binding assays, such as ELISAs and western blot than precipitin tests, and mainly for the inactivation of carbohydrate epitopes present in our antigenic preparation that led to increased WB test sensitivity [18, 26]. WB could be useful as conclusive method of laboratorial diagnosis of this mycosis.

Although, the WB showed a high sensitivity, false negative results were found in six HIV-patients with confirmed disseminated histoplasmosis. This mycosis usually occurs in HIV-patients that have advanced immunosuppression [34, 35]. Probably, the absence of detectable antibodies may be associated with this event. The time of serum collection is also an important variable in the detection of antibodies in histoplasmosis since anti-M and anti-H antibodies are detected between 2 and 6 weeks, and posteriorly formation of immune complexes [30].

Reactivity against the M band (88 kDa), was also observed in 7 false-positive serum samples; five sera from patients with paracoccidiodomycosis, one with aspergillosis, and one with tuberculosis, similar to previously results [18]. However, antibodies against M band can persist for long time after disease resolution [3] without clinical manifestations compatible with histoplasmosis. For instance, in individuals from endemic areas, such as Rio de Janeiro state, the presence of these antibodies can represent only previous exposition to *H. capsulatum*. In these cases, the results should be carefully interpreted, and in this way, does not lead to a mistaken diagnosis of histoplasmosis in patients affected with other infectious diseases [33]. However, the association of clinical and epidemiological data with the laboratory evidences address in favor of the reliability of the test. In addition, co-infections with other fungal infections or granulomatous lung diseases such as tuberculosis may coexist in these patients [36]. This event also may usually occur in individuals with severe immunosuppression or in those that suffer of other pulmonary disease [37, 38].

The accuracy is an essential parameter for the validation of a diagnostic test, and it is characterized by reproducibility measurements of the test. In this study we observed intra- and interobserver agreement in the WB test, which classify the test as almost perfect, making it possible to be applied in multicenter studies.

It has been demonstrated that nitrocellulose membrane is optimal for transfer and subsequent binding of specific proteins. We previously demonstrated that western blot using nitrocellulose membranes probed with ptHMIN was an excellent method to detect antibodies in patients infected with *H. capsulatum* [18, 25, 26]. However, up to date, there is not information about the time that the nitrocellulose membranes, sensitized with ptHMIN antigen, can remain viable and without structural damage for the use in WB [18, 25, 26]. Our study demonstrated that WB sensitized strips may be stored at room temperature up to 5 years, presenting reactivity in WB without compromising test quality. With this, there is a potential for pre-manufactured blot strips to simplify test performance, permitting the distribution to other laboratories in order to promote a worldwide multicenter evaluation.

Future studies of WB validation will be conducted in different Brazilian regions in order to evaluate a large number of samples. Furthermore, additional evaluation of this assay is needed to better characterize its clinical sensitivity in untreated and treated patients, the kinetics of antibody clearance as measured by this test, and the ability of antibody tests in general to predict treatment success or failure, mainly in the acute form of histoplasmosis. Importantly, the validated test is simple to perform, requiring only a basic lab infrastructure for its realization. This method can be performed by any professional, if properly trained and special attention should be given to reading the test result.

## Conclusion

The WB for diagnosis of histoplasmosis, using ptHMIN antigen, showed that this method can be considered a useful tool in the diagnosis of histoplasmosis being used by public health system, even in situations where laboratory facilities are relatively limited. In addition, WB is faster than those test used in diagnostic routine, and should be applied in microbiology laboratories since has almost perfect reproducibility, producing repeated and consistent results. Cost evaluations are necessary to completely define the role of this technique on large scale. As future perspective, multicenter trials should be held involving laboratories from other Brazilian regions or even other countries engaged in the diagnosis of this mycosis.
